# Supplementary material for: Similarity Constrained CC2: Toward Efficient Coupled Cluster Nonadiabatic Dynamics among Excited States
Source: J Chem Theory Comput. 2025 Oct 15;21(20):10466–73. doi: 10.1021/acs.jctc.5c00997 (PMC12573744; doi:10.1021/acs.jctc.5c00997)
Supplement: Supplementary file 1 [file ct5c00997_si_001.pdf]

Supplementary Information

Similarity Constrained CC2: Toward Efficient Coupled Cluster Nonadiabatic Dynamics Among Excited States

Leo Stoll, Sara Angelico, Eirik F. Kjønstad, and Henrik Koch*

Department of Chemistry, Norwegian University of Science and Technology, 7491 Trondheim, Norway

E-mail: henrik.koch@ntnu.no

Contents

1 Derivations **S2**

2 Implementation-ready Expressions for SCC2 **S4**

3 CI Geometry for HOF **S6**

4 Geometries for 2D-scan of Thymine **S7**

5 Size-scaling properties **S9**

References **S11**

1 Derivations

We have defined the T1-transformed Hamiltonian $\bar{H}$ as[1]

$$\bar{H} = \exp(-\hat{T}_1)\hat{H}\exp(\hat{T}_1) = \bar{F} + \bar{U} \tag{1}$$

$$\bar{F} = \exp(-\hat{T}_1)\hat{F}\exp(\hat{T}_1) \tag{2}$$

$$\bar{U} = \exp(-\hat{T}_1)\hat{U}\exp(\hat{T}_1) \tag{3}$$

and apply the Fock-operators commutators with general excitation operators $\hat{\tau}_\mu, \hat{\tau}_\nu$[1]

$$[\bar{F}, \hat{\tau}_\mu] = \epsilon_\mu \hat{\tau}_\mu \tag{4a}$$

$$[[\bar{F}, \hat{\tau}_\mu], \hat{\tau}_\nu] = 0 \tag{4b}$$

Then, using the SCC2 cluster operator $\hat{T} = \hat{T}_1 + \hat{T}_2 + \zeta \hat{X}_3$ where we consider $\hat{X}_3$ to be of first order in the fluctuation potential, and with the perturbative truncations outlined in the article, we obtain the following terms for the SCC2 amplitude equations:

$$\begin{aligned}
\Omega_{\mu_1} &= \langle \mu_1 | \exp(-\hat{T}_2 - \hat{X}_3)\, \bar{H}\, \exp(\hat{T}_2 + \hat{X}_3) | \mathrm{HF} \rangle \\
&= \langle \mu_1 | \bar{H} + \underbrace{\cancel{[\bar{F}, \hat{T}_2]}}_{\text{operator rank}} + [\bar{U}, \hat{T}_2] + \underbrace{\cancel{[\bar{F}, \hat{X}_3]}}_{\text{operator rank}} + [\bar{U}, \hat{X}_3] | \mathrm{HF} \rangle \\
&= \langle \mu_1 | \bar{H} + [\bar{U}, \hat{T}_2] + [\bar{U}, \hat{X}_3] | | \mathrm{HF} \rangle
\end{aligned} \tag{5}$$

$$
\begin{aligned}
\Omega_{\mu_2} &= \langle \mu_2 | \exp(-\hat{T}_2 - \hat{X}_3)\, \bar{H} \exp(\hat{T}_2 + \hat{X}_3) | \mathrm{HF} \rangle \\
&= \langle \mu_2 | \bar{H} + [\bar{F}, \hat{T}_2] + \underbrace{\cancel{[\bar{U}, \hat{T}_2]}}_{2^{\mathrm{nd}} \text{ order in } U} + \underbrace{\cancel{[\bar{F}, \hat{X}_3]}}_{\text{operator rank}} + \underbrace{\cancel{[\bar{U}, \hat{X}_3]}}_{2^{\mathrm{nd}} \text{ order in } U} \\
&\quad + \underbrace{\cancel{\frac{1}{2}[[\bar{F}, \hat{T}_2], \hat{T}_2]}}_{Eq.(4)} + \underbrace{\cancel{\frac{1}{2}[[\bar{U}, \hat{T}_2], \hat{T}_2]}}_{3^{\mathrm{rd}} \text{ order in } U} + \underbrace{\cancel{[[\bar{F}, \hat{T}_2], \hat{X}_3]}}_{Eq.(4)} + \underbrace{\cancel{[[\bar{U}, \hat{T}_2], \hat{X}_3]}}_{\substack{\text{operator rank} \\ 3^{\mathrm{rd}} \text{ order in } U}} \\
&\quad + \underbrace{\cancel{\frac{1}{2}[[\bar{F}, \hat{X}_3], \hat{X}_3]}}_{Eq.(4)} + \underbrace{\cancel{\frac{1}{2}[[\bar{U}, \hat{X}_3], \hat{X}_3]}}_{\substack{\text{operator rank} \\ 3^{\mathrm{rd}} \text{ order in } U}} | \mathrm{HF} \rangle \\
&= \langle \mu_2 | \bar{H} + [\bar{F}, \hat{T}_2] | \mathrm{HF} \rangle .
\end{aligned}
\tag{6}
$$

2 Implementation-ready Expressions for SCC2

The T1-transformed Hamiltonian of Eq. (1) can be expressed as the usual electronic Hamiltonian using the singlet operator, but with the T1-transformed one- and two electron integrals, respectively $\bar{h}_{pq}$ and $\bar{g}_{pqrs}$[1]

$$\begin{aligned}\bar{H} = &\sum_{pq} \bar{h}_{pq} E_{pq} \\ &+ \frac{1}{2} \sum_{pqrs} \bar{g}_{pqrs} \left(E_{pq} E_{rs} - \delta_{qr} E_{ps} \right) + h_{\text{nuc}}\end{aligned} \tag{7}$$

where $p, q, r, s \ldots$ are MO indices. Notably, the transformed two-electron integrals have reduced symmetry with respect to the non T1-transformed two-electron integrals, maintaining only particle symmetry $\bar{g}_{pqrs} = \bar{g}_{rspq}$.

Using the T1-transformed integrals, the SCC correction to the singles amplitude equations, $\Omega^{\text{SCC}}_{\mu_1}$, can be written on integral form as[2]

$$\Omega^{\text{SCC}}_{ai} = \sum_{bjck} \left(x^{abc}_{ijk} - x^{abc}_{kji} \right) \bar{L}_{jbkc} \tag{8}$$

with

$$x^{abc}_{ijk} = \zeta P^{-AB} \left(r^{A}_{ai} \tilde{r}^{B}_{bjck} + r^{A}_{bj} \tilde{r}^{B}_{aick} + r^{A}_{ck} \tilde{r}^{B}_{aibj} \right) \tag{9}$$

$$\bar{L}_{pqrs} = 2\bar{g}_{pqrs} - \bar{g}_{psrq} \tag{10}$$

$$\tilde{r}^{k}_{aibj} = r^{k}_{aibj} (1 + \delta_{ai,bj}) \tag{11}$$

$$P^{-AB} f(A, B) = f(A, B) - f(B, A). \tag{12}$$

The orthogonality condition can be calculated using the following expressions for $\boldsymbol{q}$

$$q_{ai} = t^{a}_{i} \qquad q_{aibj} = \frac{1}{1 + \delta_{ai,bj}} (t^{ab}_{ij} + t^{a}_{i} t^{b}_{j}) \tag{13}$$

and the following matrix transformations of a vector $\boldsymbol{c}^2$

$$(\boldsymbol{Q}\boldsymbol{c})_{ai} = c_{ai} \tag{14a}$$

$$(\boldsymbol{Q}\boldsymbol{c})_{aibj} = c_{aibj} + \frac{1}{1+\delta_{ai,bj}}(c_{ai}t_j^b + c_{bj}t_i^a) \tag{14b}$$

$$(\boldsymbol{c}^T\boldsymbol{Q}^T)_{ai} = c_{ai} + \sum_{ck} t_k^c b_{aick} \tag{15a}$$

$$(\boldsymbol{c}^T\boldsymbol{Q}^T)_{aibj} = c_{aibj} \tag{15b}$$

$$(\boldsymbol{\mathcal{S}}\boldsymbol{c})_{ai} = 2c_{ai} \tag{16a}$$

$$(\boldsymbol{\mathcal{S}}\boldsymbol{c})_{aibj} = 2(1+\delta_{ai,bj})(2c_{aibj} - c_{ajbi}). \tag{16b}$$

3 CI Geometry for HOF

The conical intersection between the states 1^1A' and 2^1A' of HOF for an OH bond length of 1.1 Å was located by scanning the 2D plane spanned by the remaining internal coordinates. It was encountered at $R_{\mathrm{OF}} = 1.3321938$Å and $\theta_{\mathrm{HOF}} = 90.2475°$. This corresponds to the following geometry in cartesian coordinates:

Table S1: Geometry for SCC2/aug-cc-pVDZ conical intersection between the states 1^1A' and 2^1A' of HOF for an OH bond length of 1.1 Å. Geometry provided in Angstrom.

Atom	x	y	z
H	-0.004750684189	1.099989741316	0.000000000000
O	0.000000000000	0.000000000000	0.000000000000
F	2.517481426844	0.000000000000	0.000000000000

4 Geometries for 2D-scan of Thymine

The basis vectors used to span the gh-plane used in the 2D-scan of thymine were obtained from the g- and h-vectors available from SCCSD calculations of a minimum energy conical intersection (MECI) in a distorted (non-planar) geometry.[3] The geometry for the S_1/S_2 SCC2 conical intersection was located within the branching plane of this SCCSD MECI.

Table S2: Geometry for SCC2/cc-pVDZ conical intersection between states S_1 and S_2 located in thymine 2D-scans. Geometry provided in bohr.

Atom	x	y	z
H	3.595889643580	3.282432604151	0.975922146276
H	-1.154079844480	4.850251870840	-0.995000020486
H	2.184193690383	-3.852070634671	-0.143708705776
H	-5.183882375697	3.738832874934	1.242990457532
H	-6.200931791418	0.101331649512	0.678594175727
H	-5.493251220592	2.111726044220	-1.734813526935
C	3.071302454392	-0.159842689030	-0.013548339899
C	-0.403787069982	3.023873528098	-0.362747000370
C	-1.529548654373	-1.596338517454	-0.154071584974
C	-2.203312576980	1.062221681888	-0.012296553478
C	-4.981786031830	1.811395162821	0.241722276629
N	1.186491679583	-1.904180617241	-0.153756142661
N	2.083083366355	2.288810152087	0.107079474525
O	5.307053702028	-0.536037647085	0.107398024976
O	-2.928617287789	-3.497304711432	0.108434295250

To obtain a suitable depiction of the conical intersection, first the h-vector was orthogonalized with respect to the g-vector. Then both vectors were normalized, upon which they were rotated counterclockwise by three degrees in the plane they span, $v = \cos 3° g + \sin 3° h$ and $w = -\sin 3° g + \cos 3° h$, where v corresponds to g and w corresponds to h as labeled in the article. Finally, v and w were rescaled as to produce a nearly circular conical intersection in CC2/cc-pVDZ calculations. The final vectors v and w are given below.

Table S3: v-vector used to span the gh-plane in thymine 2D-scans.

Atom	x	y	z
H	-0.00910526	-0.01966916	-0.00470308
H	-0.01248047	0.00582823	-0.00378694
H	-0.0169608	0.05170705	0.00113383
H	0.00284974	0.01112344	-0.00131754
H	-0.01329086	-0.02306126	-0.00640475
H	0.00267549	-0.00649999	-0.00127202
C	-0.01362692	-0.01236836	-0.00871645
C	0.00787804	-0.00810476	-0.0044191
C	-0.01722977	0.01494915	-0.00565207
C	-0.00594766	0.00842545	-0.00216768
C	0.01153683	0.02004446	0.01598482
N	0.05726479	-0.05634807	0.00546124
N	0.00775673	0.02887218	0.01335076
O	0.00602834	-0.00351277	0.00137911
O	-0.0073482	-0.01138553	0.00112988

Table S4: w-vector used to span the gh-plane in thymine 2D-scans.

Atom	x	y	z
H	-1.64401523e-04	-4.75539068e-05	-5.23155035e-05
H	-6.29998517e-05	-6.42700521e-05	1.10981744e-04
H	1.89669016e-05	-9.62072649e-05	5.21042299e-06
H	8.00721628e-06	-9.03302695e-05	2.73396897e-05
H	-4.74427001e-05	8.57998386e-05	2.20447142e-05
H	-1.01652755e-04	6.71906680e-06	8.44935763e-05
C	1.95697650e-04	1.46616735e-03	9.33302245e-05
C	-8.25662194e-04	-7.64532457e-04	3.77165417e-04
C	1.39151644e-03	2.23063732e-03	-2.88562194e-04
C	3.22753138e-04	3.65827178e-04	-5.12915698e-05
C	-2.16299607e-04	-2.52840985e-06	-4.05688216e-04
N	-4.13791871e-04	-6.65569635e-04	3.46923635e-05
N	8.83866255e-04	-5.10535001e-04	-2.51239393e-04
O	-2.11635332e-04	-4.33079509e-04	-2.25471634e-05
O	-7.76911146e-04	-1.48050989e-03	3.16392145e-04

5 Size-scaling properties

SCC2 excitation energies for the lowest four excited states of HOF with varying numbers of surrounding argon atoms are provided in Table S5. Up to the convergence threshold of 10^{-5} Hartree, no changes in the excitation energies are observed. This indicates that size-intensivity is conserved at least up to this accuracy. Similarly, total ground state energies for the same set of systems are provided in Table S6, together with expected values given calculations of a free HOF molecule and a free argon atom. The expected and actual values again match, up to the convergence threshold of 10^{-5} Hartree and considering error propagation when calculating the expected value from the calculation of the single HOF molecule and argon atom. This indicates that also size-extensivity is maintained up to the accuracy of the calculations.

Table S5: SCC2/cc-pVTZ excitation energies (in Hartree) for hypofluorous acid surrounded by 0-8 argon atoms situated at non-interacting distances ($>$ 50 Å) from the HOF molecule. The 1^1A' and 2^1A' states of HOF were selected as the similarity constrained states (corresponding to states 3 and 4) and the HOF geometry is defined by $R_{\mathrm{OH}} = 1.1$Å, $R_{\mathrm{OF}} = 1.33$Å, $\theta_{\mathrm{HOF}} = 90.5°$ and is outside the intersection region. All calculations were converged to a residual threshold of 10^{-5}.

#Ar	ω_1	ω_2	ω_3	ω_4
0	0.24269	0.24931	0.32132	0.33181
1	0.24269	0.24931	0.32132	0.33181
2	0.24269	0.24930	0.32132	0.33181
3	0.24269	0.24930	0.32131	0.33181
4	0.24269	0.24930	0.32131	0.33181
5	0.24269	0.24930	0.32131	0.33181
6	0.24269	0.24930	0.32131	0.33181
7	0.24269	0.24930	0.32132	0.33181
8	0.24268	0.24930	0.32132	0.33181

Table S6: SCC2/cc-pVTZ ground state energies (in Hartree) for hypofluorous acid surrounded by 0-8 argon atoms situated at non-interacting distances (> 50 Å) from the HOF molecule. The 1^1A' and 2^1A' states of HOF were selected as the similarity constrained states (corresponding to states 3 and 4) and the HOF geometry is defined by $R_{\text{OH}} = 1.1$Å, $R_{\text{OF}} = 1.33$Å, $\theta_{\text{HOF}} = 90.5°$ and is outside the intersection region. Additionally, the SCC2/cc-pVTZ ground state energy of a free HOF molecule E_{HOF} and of a free Argon atom (which corresponds to the CC2/cc-pVTZ energy) E_{Ar} are provided, together with their sum for the given numbers of Argon atoms. All calculations were converged to a residual threshold of 10^{-5}.

x	E_{HOF}	E_{Ar}	$E_{\text{HOF}} + xE_{\text{Ar}}$	$E_{\text{HOF}+x\text{Ar}}$
	-175.31908	-527.05131	-	-
1	-	-	-702.37039	-702.37040
2	-	-	-1229.42171	-1229.42171
3	-	-	-1756.47302	-1756.47303
4	-	-	-2283.52434	-2283.52437
5	-	-	-2810.57565	-2810.57570
6	-	-	-3337.62696	-3337.62702
7	-	-	-3864.67828	-3864.67835
8	-	-	-4391.72959	-4391.72967

References

(1) Helgaker, T.; Jørgensen, P.; Olsen, J. *Molecular Electronic-Structure Theory*; Wiley, 2014.

(2) Kjønstad, E. F.; Koch, H. An Orbital Invariant Similarity Constrained Coupled Cluster Model. *J. Chem. Theory Comput.* **2019**, *15*, 5386–5397.

(3) Kjønstad, E. F.; Angelico, S.; Koch, H. Coupled Cluster Theory for Nonadiabatic Dynamics: Nuclear Gradients and Nonadiabatic Couplings in Similarity Constrained Coupled Cluster Theory. *J. Chem. Theory Comput.* **2024**, *20*, 7080–7092.
